# A lightweight hyperspectral image multi-layer feature fusion classification method based on spatial and channel reconstruction

**DOI:** 10.1371/journal.pone.0322345

**Published:** 2025-05-23

**Authors:** Yuping Yin, Haodong Zhu, Lin Wei

**Affiliations:** 1 Faculty of Electrical and Control Engineering, Liaoning Technical University, Huludao, Liaoning, China; 2 The Department of Basic Education, Liaoning Technical University, Huludao, Liaoning, China; Newcastle University, UNITED KINGDOM OF GREAT BRITAIN AND NORTHERN IRELAND

## Abstract

Hyperspectral Image (HSI) classification tasks are usually impacted by Convolutional Neural Networks (CNN). Specifically, the majority of models using traditional convolutions for HSI classification tasks extract redundant information due to the convolution layer, which makes the subsequent network structure produce a large number of parameters and complex computations, so as to limit their classification effectiveness, particularly in situations with constraints on computational power and storage capacity. To address these issues, this paper proposes a lightweight multi-layer feature fusion classification method for hyperspectral images based on spatial and channel reconstruction (SCNet). Firstly, this method reduces redundant computations of spatial and spectral features by introducing Spatial and Channel Reconstruction Convolutions (SCConv), a novel convolutional compression method. Secondly, the proposed network backbone is stacked with multiple SCConv modules, which allows the network to capture spatial and spectral features that are more beneficial for hyperspectral image classification. Finally, to effectively utilize the multi-layer feature information generated by SCConv modules, a multi-layer feature fusion (MLFF) unit was designed to connect multiple feature maps at different depths, thereby obtaining a more robust feature representation. The experimental results demonstrate that, compared to seven other hyperspectral image classification methods, this network has significant advantages in terms of the number of parameters, model complexity, and testing time. These findings have been validated through experiments on four benchmark datasets.

## 1 Introduction

A spectral imager captures hyperspectral remote sensing images by simultaneously imaging target objects across multiple contiguous spectral bands. As a result, HSI consists of hundreds of continuous spectral bands ranging from the visible to the infrared spectrum. These images not only acquire spatial information such as the structure, positional relationships, and shapes of objects but also contain spectral information that characterizes the physical properties of the materials. This unique “image-spectrum integration” characteristic enables hyperspectral images to play a crucial role in various fields, including environmental monitoring [1], urban planning [2], and smart agriculture [3].

When capturing images, spectral imagers are influenced by their inherent hardware limitations and environmental factors, resulting in various types of noise that affect the quality and analytical precision of hyperspectral images. With the advancement of machine learning, spatial filtering techniques such as mean filtering [4], median filtering [5], and Gaussian filtering [6] have been employed to smooth hyperspectral images, thereby reducing noise. Moreover, the noise in hyperspectral images is distributed across all bands but is often concentrated in specific directions in high-dimensional space. Aiming at this characteristic, several feature extraction methods have been proposed, such as Principal Component Analysis (PCA) [7], Linear Discriminant Analysis (LDA) [8], and Independent Component Analysis (ICA) [9]. Subsequently, more efficient dimensionality reduction techniques have also been explored, including Autoencoder [10], Locally Linear Embedding (LLE) [11], and t-distributed Stochastic Neighbor Embedding (t-SNE) [12]. However, a major drawback of dimensionality reduction is the inevitable loss of information. When reducing high-dimensional data to lower dimensions, some detailed information and spectral features may be lost, potentially affecting the accuracy of classification tasks. Therefore, during the preprocessing of hyperspectral images, it is crucial to preserve spectral information as much as possible and avoid excessive processing that leads to information loss. Zero-phase Component Analysis (ZCA) [13], an extension of PCA, can effectively improve the signal-to-noise ratio of the data while retaining all band information. ZCA achieves this by rotating the PCA-transformed data back to the original feature space, making the processed data more closely resemble the original input data.

In recent years, CNN has been extensively applied in computer vision tasks due to its remarkable multi-level feature learning capabilities. Consequently, various CNN-driven HSI classification methods have been proposed, which can be broadly categorized into 1D convolutional neural network (1DCNN) [14], 2D convolutional neural network (2DCNN) [15], 3D convolutional neural network (3DCNN) [16], and some hybrid methods. The 1D-CNN focuses on spectral feature extraction, treating each pixel’s spectral curve as a one-dimensional signal for convolution operations. The 2D-CNN emphasizes spatial feature extraction, capturing features of spatial dimension by performing convolutions on the two-dimensional image of each spectral band. The 3D-CNN conducts convolutions on the three-dimensional data cube of hyperspectral images, simultaneously capturing joint spectral and spatial features. In 2019, Roy *et al*. [17] proposed the HybridSN model, which combines 3D-CNN and 2D-CNN, leveraging the complementary information from both spatial-spectral and spectral domains for HSI classification.

However, the aforementioned algorithms based on deep convolutional neural networks rely on extensive computational and storage resources, posing significant challenges for efficient deployment in resource-constrained environments. To overcome these challenges, various network architecture designs have been explored to enhance network efficiency, aiming to reduce the inherent redundancy of model parameters and further achieve lightweight network models. For instance, ResNet [18] and DenseNet [19] employ efficient shortcut connections to improve network topology, connecting all preceding feature maps to reduce redundant parameters while alleviating the training difficulties of deep networks. Inspired by ResNet, Zhong *et al*. [20] proposed a Spectral-Spatial Residual Network (SSRN), where residual blocks are connected via identity mappings to every other three-dimensional convolution layer, facilitating gradient backpropagation and reducing model parameters while mitigating accuracy degradation. Li *et al*. [21] introduced DenseNet into the Dual-Branch Dual-Attention Mechanism Network (DBDA), designing dense spectral blocks and dense spatial blocks to learn deeper spectral and spatial features of hyperspectral images. The densely connected arrangement of dense blocks deepens the network, reduces gradient vanishing, and effectively compresses the model. Xue *et al*. [22] designed a novel Spectral-Spatial Siamese Network (S3Net). This network consists of a lightweight Spectral-Spatial Network (SSN) composed of one-dimensional and two-dimensional convolutions to extract spectral-spatial features. They constructed a dual-branch SSN, which expands the training set by inputting sample pairs into each branch, thereby improving classification performance in small sample scenarios.

To further reduce model parameters and FLOPs, researchers often use various efficient convolution operations to replace traditional convolutions. For example, MADANet [23] utilizes depth-wise separable convolutions to extract and aggregate multi-scale features, effectively capturing local contextual information and achieving outstanding classification accuracy with fewer parameters. GhostNet [24], considering redundancy among feature maps, generates primary feature maps using a small number of standard convolutions and produces multiple ghost feature maps through simple linear transformations (such as pointwise and depth-wise convolutions). This approach reduces the computational cost of standard convolutions while maintaining high representational capability. Similarly, OctConv [25] introduces octave convolution, dividing input features into high-frequency and low-frequency channels, where the latter are processed with reduced spatial resolution to alleviate spatial redundancy, cutting computation without increasing the parameter count. However, the aforementioned methods either focus on reducing redundancy in the channel dimension or the spatial dimension, leaving the network still facing the problem of feature redundancy.

Inspired by previous models aimed at reducing the number of parameters and computational costs of convolutional layers, researchers have proposed a novel CNN compression method known as Spatial and Channel Reconstruction Convolutions (SCConv) [26]. This module consists of Spatial Reconstruction Units (SRU) and Channel Reconstruction Units (CRU). SRU separates and reconstructs features based on weights to suppress spatial redundancy and enhance feature representation. CRU employs split, transformation, and fusion strategies to reduce channel redundancy while simultaneously lowering computational costs and storage requirements. By sequentially arranging SRU and CRU to replace standard convolutions, the combined approach reduces both spatial and channel redundancy in convolutional layers, significantly cutting computational costs and enhancing the performance of deep models.

Harnessing these recent technological advancements, this paper proposes a novel lightweight HSI classification network architecture. In this approach, the original HSI data is first preprocessed using ZCA, which retains all band information and reduces the impact of noise in the raw data. Subsequently, by introducing SCConv to replace standard convolutions, the redundancy in the spatial and spectral dimensions of intermediate feature maps is reduced, leading to a decrease in the number of parameters and computational complexity. Additionally, to fully exploit the multiscale differences of feature maps at different depths, a multi-layer feature fusion (MLFF) unit was proposed to generate more representative features. Finally, experimental results demonstrate the effectiveness of the proposed network architecture.

In general, the main contributions of this article can be enumerated as follows:

(1) This paper proposes a lightweight network structure with fewer layers for overall design. This structure maintains considerable classification performance with a reduced number of parameters and lower computational costs.

(2) To overcome the drawbacks of spatial and channel redundancy produced by conventional convolutional layers, this study introduces SCConv, composed of SRU and CRU. This not only significantly reduces computational costs but also enhances the performance of deep models.

(3) Due to the presence of various feature information at different layers of SCNet, a MLFF unit were designed to fuse these features from different depths. The proposed MLFF unit further improves the classification accuracy of the network.

## 2 Related works

### 2.1 Group normalization

Group normalization (GN) [27] is a novel normalization technique in deep learning. Compared to batch normalization (BN) [28], GN is independent of batch size, leading to enhanced stability across varying batch sizes. BN normalizes each channel dimension across the entire batch, calculating the mean and variance along this dimension. Suppose BN was applied to a data set $X\in {\mathbb{R}}^{N\;\times \;C\;\times \;H\;\times \;W}$, where N is the batch size, C is the channel dimension, and H and W are the height and width. For each channel i, BN computes the mean μ and variance $\sigma^{2}$ along the (N, H, W) axes as follows:

$$\mu_{i}=\frac{1}{N\times H\times W}\sum_{n=1}^{N}\sum_{h=1}^{H}\sum_{w=1}^{W}x_{n,i,h,w}$$ (1)

$$\sigma^{2}=\frac{1}{N\times H\times W}\sum_{n=1}^{N}\sum_{h=1}^{H}\sum_{w=1}^{W}{\left(x_{n,i,h,w}-\mu_{i}\right)}^{2}$$ (2)

The calculation of mean and variance in GN differs from that in BN. GN first divides the channels into multiple groups and normalizes the features within each group. Consequently, the mean and variance are computed based on the features within each group. If the channels of X are divided into G groups, with each group containing C/G channels, the mean $\mu_{i}$ and variance $\sigma^{2}$ are calculated as follows:

$$\mu_{i}=\frac{1}{\frac{C}{G}\times H\times W}\sum_{g=1}^{\frac{C}{G}}\sum_{h=1}^{H}\sum_{w=1}^{W}x_{i,g,h,w}$$ (3)

$$\sigma^{2}=\frac{1}{\frac{C}{G}\times H\times W}\sum_{g=1}^{\frac{C}{G}}\sum_{h=1}^{H}\sum_{w=1}^{W}{\left(x_{i,g,h,w}-\mu_{i}\right)}^{2}$$ (4)

**Fig 1:**
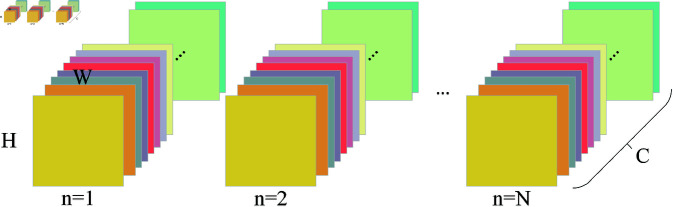
Batch normalization.

**Fig 2:**
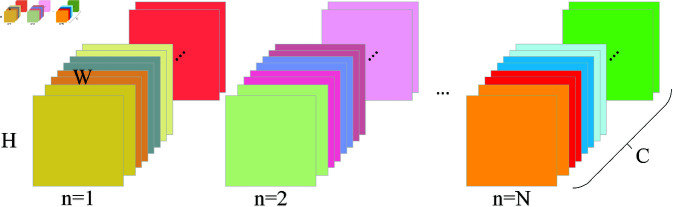
Group normalization (*g* = 2).

For the data X, normalization can be performed by subtracting the mean μ and dividing by the standard deviation σ, as shown below:

$$X_{out}=\gamma \frac{X-\mu }{\sigma +\varepsilon }+\beta$$ (5)

where, μ and σ are the mean and standard deviation of X, ε is a tiny constant added for division stability, and γ and β are trainable parameters. The illustrations of BN and GN with *g* = 2 are shown in Figs 1 and 2. The μ and σ are computed from the pixel values within the same color feature map.

Additionally, there are two normalization methods similar to GN in terms of computation: Layer Normalization (LN) and Instance Normalization (IN). Their main difference lies in the dimension over which normalization is performed. LN normalizes along the channel dimension (*C*) of a single sample, computing the mean and variance across all pixels within the entire channel. In contrast, IN normalizes each channel separately at the spatial level (H×W), computing the mean and variance independently for each channel. GN falls between LN and IN, with its normalization scope determined by the number of groups G. When *G* = 1, GN degrades to LN, performing normalization across the entire channel dimension. Conversely, when *G* = *C*, GN becomes equivalent to IN, normalizing each channel independently.

In terms of normalization relationships, LN, IN, and GN are all batch-independent normalization methods. Unlike BN, which requires computing the mean and variance across multiple samples, these methods are more adaptable to small-batch training environments. They help avoid the issue of unstable statistics in BN during small-batch training, thereby improving model stability and generalization in memory-constrained or dynamically changing input scenarios.

### 2.2 PCA and ZCA

**Fig 3:**
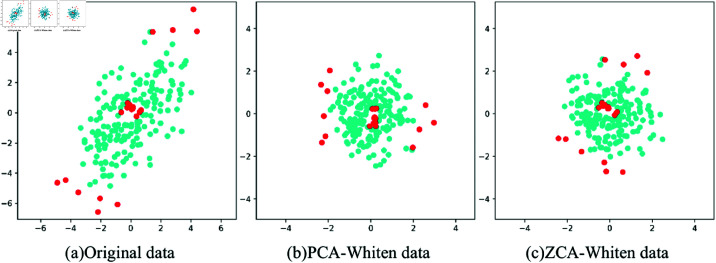
Visualization of whitening effectiveness. (a) Original data. (b) PCA whiten data. (c) ZCA whiten data.

Principal Component Analysis (PCA) [13] is one of the most commonly used and effective dimensionality reduction algorithms. PCA processes all the bands in the original image, makes them pairwise orthogonal, extracts their main features, and then transforms them into a new feature space and makes them maintain the original information as much as possible when reflecting the spectral information to ensure the accuracy of subsequent training. PCA whitening is an extension of PCA. The difference from PCA is that after making the bands pairwise orthogonal, the variance of each band feature is set to 1, and then the subsequent operations of PCA are performed. This prevents the model from being biased towards certain features during training and makes it more fair and balanced. Zero-phase Component Analysis (ZCA) [13] whitening rotates the PCA-whitened data back to the original feature space on the basis of PCA whitening. In the case of two-dimensional data, the original data, the data after PCA whitening, and the data after ZCA whitening are shown in Fig 3. It is worth noting that ZCA whitening is not a dimensionality reduction algorithm; instead, it retains all the band features, making the transformed data closer to the original input data. The ZCA algorithm process is as follows:

**Algorithm 1** ZCA

**Input:** $X\in {\mathbb{R}}^{n\times m}$

**Output:** $X_{ZCAWhiten}\in {\mathbb{R}}^{n\times m}$

1: $\tilde{X}\leftarrow X-\mu$ //Zero Mean Normalization

2: $\Sigma \leftarrow \frac{1}{m-1}\tilde{X}{\tilde{X}}^{T}$

3: $\Sigma \leftarrow U\Lambda U^{T}$ //Orthogonal Decomposition

4: $X_{rotate}\leftarrow U^{T}X$

5: $X_{PCAWhiten}\leftarrow \Lambda^{\frac{1}{2}}X_{rotate}$

6: $X_{ZCAWhiten}\leftarrow UX_{PCAWhiten}$

## 3 Methodology

### 3.1 Proposed model architecture for HSI classification

**Fig 4:**
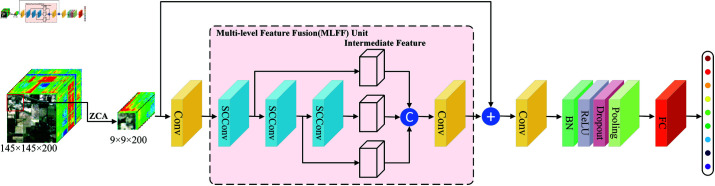
The architecture of proposed SCNet.

The overall network structure of SCNet is illustrated in Fig 4. Firstly, ZCA whitening is used to eliminate the correlation between the bands of the original HSI. Subsequently, the whitened data is divided into smaller 3D patches, and each is labeled with the ground truth of the central pixel of the 3D patch. In this paper, the Indian Pines dataset is used, with the spatial size of the blocks set to 9 as the input for the entire network.

As observed in Fig 4, the proposed network implements a straightforward backbone unit that first uses a 1×1 convolution to expand the dimensionality of the input 3D patch, to satisfy the channel requirements for subsequent SCConv layers. By employing more convolutional kernels, the network can comprehensively extract features, thereby better learning the critical information from the data. Then, by stacking three SCConv modules, the spatial and channel redundancies within the 3D patches are reduced. To fully utilize the features from different depths, this paper designed the MLFF unit to connect multiple feature maps along the spectral dimension. This MLFF unit not only stabilizes the network but also makes it easier to train. Furthermore, by using 1×1 convolution to compress the fused feature maps, high-level HSI features are extracted, and redundant information is reduced. Ultimately, all features are collected and vectorized by a final 3×3 convolutional and pooling block before the output is sent to the classifier, which is a fully connected (FC) multilayer perceptron (MLP) layer. It should be noted that instead of direct prediction, global residual learning was used to construct the output, which can provide a smoother hypersurface for gradient descent in SCNet and effectively reduce the risk of network degradation. Additionally, the number of bottlenecks utilized for the HSI classification network is greatly decreased by our suggested network topology, which avoids data degradation and gradient vanishing problems during forward and backward propagation in addition to reducing model overfitting.

### 3.2 The architecture of SCConv

**Fig 5:**
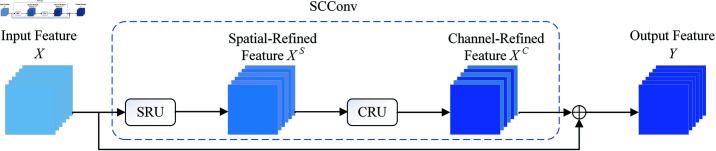
The structure of SCConv with residual connection.

The SCConv module, depicted in Fig 5, comprises two units: Spatial Reconstruction Unit (SRU) and Channel Reconstruction Unit (CRU). Specifically, the SCConv module uses the SRU operation to obtain the spatial-refined features *X^S^* for the input features *X* in the bottleneck residual block. Subsequently, the channel-refined features *X^C^* are obtained using the CRU operation. This approach utilizes the spatial redundancy and channel redundancy available in the features within SCConv module. As a result, it decreases redundant information among intermediate feature maps and improves the feature representation capabilities of CNN.

#### 3.2.1. SRU for spatial redundancy.

**Fig 6:**
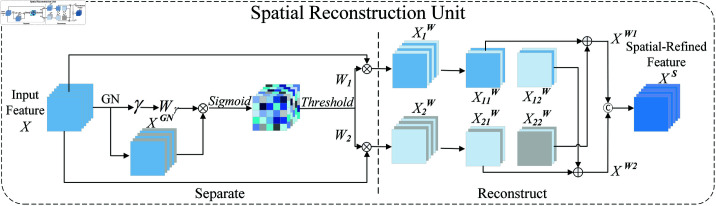
The architecture of spatial reconstruction unit.

To utilize the spatial redundancy of features, SCConv module designs Spatial Reconstruction Unit (SRU) shown in Fig 6, which consists of two steps: Separate and Reconstruct.

**Separate:** The objective of the separation operation is to separate feature maps which have abundant spatial information from feature maps which have less information. Firstly, let’s use the following equation:

$$X^{GN}=GN\left(X\right)=\gamma \frac{X-\mu }{\sigma +\varepsilon }+\beta$$ (6)

For a given intermediate feature map $X\in {\mathbb{R}}^{N\times C\times H\times W}$, GN is applied, and the trainable parameters γ and β in GN are used to evaluate the richness of information in different feature maps. Richer spatial information reflects more variations in spatial pixels, leading to higher values of γ. By normalizing γ, the weight $W_{\gamma }\in {\mathbb{R}}^{C}$ can be obtained that indicates the richness of information in different feature maps [26].

$$W_{\gamma }=\left\{w_{i}\right\}=\frac{\gamma_{i}}{\sum_{j=1}^{C}\gamma_{i}},i,j=1,2,\ldots ,C$$ (7)

Then, the GN feature maps are re-weighted by $W_{\gamma }$, and a Sigmoid function is used to map the values to the (0, 1) range. Subsequently, a threshold is set for gating (the experiments set the threshold to 0.5). Weights exceeding the threshold are set to 1 to obtain the rich information weights *W*_1_, while weights below the threshold are set to 0 to obtain the sparse information weights *W*_2_. The entire process of obtaining W can be expressed by the following equation, where ⊗ is element-wise multiplication [26],

$$W=Gate\left(Sigmoid\left(W_{\gamma }⊗X^{GN}\right)\right)$$ (8)

Finally, the input feature X is multiplied with *W*_1_ and *W*_2_ respectively, resulting in two weighted features: the informative feature $X_{1}^{w}$ and the less informative feature $X_{2}^{w}$. $X_{1}^{w}$ contains rich and expressive spatial content, while $X_{2}^{w}$ has little or no information and is regarded as redundant content.

**Reconstruct:** To reduce the spatial redundancy, a further reconstruction operation is proposed, which adds the rich informative features to the less informative ones, generating features with more informative and occupy less spatial. Note that rather than directly adding the two parts of the features, a cross-reconstruction operation is used to fully combine the two weighted features with different information and enhance the information flow between them. Finally, the cross-reconstructed features $X_{1}^{w}$ and $X_{2}^{w}$ are concatenated together to form the spatial-refined feature maps *X^S^*. The entire reconstruction process can be expressed as follows:

$$\{X_{1}^{w}=W_{1}⊗X\;X_{2}^{w}=W_{2}⊗X\;X_{11}^{w}⊕X_{22}^{w}=X^{w1}\;X_{21}^{w}⊕X_{12}^{w}=X^{w2}\;X^{w1}⋃X^{w2}=X^{S}$$ (9)

where ⊗ is element-wise multiplication, ⊕ is element-wise summation, and ⋃ is concatenation. By applying the SRU on the input feature X, not only are the rich information features separated from the less informative ones, but their representative characteristics are also enhanced through the reconstruction operation, suppressing redundancy in the spatial dimension. Nevertheless, the spatial-refined feature maps *X^S^* still contain redundancy along the channel dimension.

#### 3.2.2 CRU for channel redundancy.

**Fig 7:**
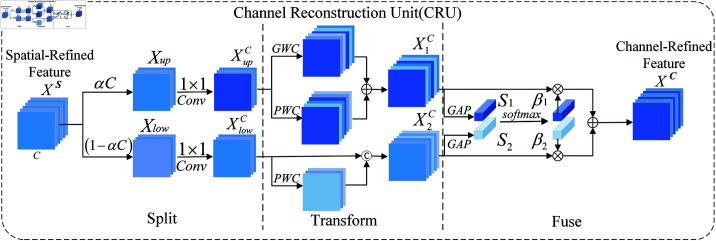
The architecture of channel reconstruction unit.

To utilize the channel redundancy of features, the SCConv module introduces the Channel Reconstruction Unit (CRU) shown in Fig 7, which applies Split, Transform, and Fuse strategies.

**Split:** For a given spatial-refined feature *X^S^*, CRU first splits *X^S^* into two parts along the channel dimension, containing αC channels and (1−α)C channels, respectively, as illustrated in the split section of Fig 7, where, α represents the split ratio (α is set to 0.5 to better balance performance and efficiency). Subsequently, 1×1 convolutions are further employed to reduce the channels of the feature map to improve computational efficiency. Here, a compression ratio *r* is introduced to control the number of feature channels to balance the computational cost of the CRU (in the experiments, *r* is set to 2). After the split and compression operations, the spatial-refined feature *X^S^* is divided into an upper part $X_{up}^{C}$ and a lower part $X_{low}^{C}$.

**Transform:** $X_{up}^{C}$ is input to the upper transformation stage and the operations of k×k GWC (the group size g was assigned to 2 in this study) and 1×1 PWC are used for $X_{up}^{C}$, respectively. Then, the outputs are summed to form a merged representative feature map $X_{1}^{C}$, as shown in the transformation part of Fig 7. By employing efficient convolution operations (i.e., GWC and PWC) instead of the costly standard 3×3 convolution, it can not only extract high-level representative information, but also reduce the computational cost. GWC decreases the number of parameters and computational requirements due to sparse convolutional connections, but cuts off the information flow between different channel groups. PWC compensates for the information loss and helps the information transfer between the channel groups. The upper transformation stage can be expressed as [26]:

$$X_{1}^{C}=W_{G}\cdot X_{up}^{C}+W_{P1}\cdot X_{up}^{C}$$ (10)

where $W_{G}\in {\mathbb{R}}^{\frac{\alpha C}{r}\;\times \;k\;\times \;k\;\times \;C}$ and $W_{P1}\in {\mathbb{R}}^{\frac{\alpha C}{r}\;\times \;1\;\times \;1\;\times \;C}$ denote the learnable weight matrices for GWC and PWC, and $X_{up}^{C}\in {\mathbb{R}}^{\frac{\alpha C}{r}\;\times \;h\;\times \;w}$ and $X_{1}^{C}\in {\mathbb{R}}^{C\;\times \;h\;\times \;w}$ are the upper input feature map and output feature map, respectively. In short, the upper transformation stage utilizes the combination of GWC and PWC on the same feature map $X_{up}^{C}$ to extract rich representative features with less computational cost.

$X_{low}^{C}$ is fed into the lower transformation stage, where an economical 1×1 PWC operation is employed to generate feature maps containing shallow hidden details. Additionally, the feature $X_{low}^{C}$ is reused to obtain more feature maps without incurring extra costs. Finally, the generated and reused feature maps are concatenated to form the output of the lower stage $X_{1}^{C}$, as follows [26]:

$$X_{2}^{C}=W_{P2}\cdot X_{low}^{C}⋃{\;X}_{low}^{C}$$ (11)

where $W_{P2}\in {\mathbb{R}}^{\frac{\left(1-\alpha \right)C}{r}\;\times \;1\;\times \;1\;\times \;\left(1-\frac{1-\alpha }{r}\right)C}$ represents the learnable weight matrix for PWC, ⋃ denotes the concatenation operation, and $X_{low}^{C}\in {\mathbb{R}}^{\frac{\left(1-\alpha \right)C}{r}\;\times \;h\;\times \;w}$ and $X_{2}^{C}\in {\mathbb{R}}^{C\;\times \;h\;\times \;w}$ are the input and output feature maps of the lower transformation stage, respectively. In summary, the lower transformation stage reuses the preceding feature $X_{low}^{C}$ and employs the economical 1×1 PWC to obtain supplementary detailed features $X_{2}^{C}$.

**Fuse:** After the transformation, the two types of features are not directly concatenated or combined. Instead, the output features from the upper and lower transformation stages, $X_{1}^{C}$ and $X_{2}^{C}$, are adaptively fused, as illustrated in the fusion part of Fig 7. First, global average pooling (GAP) is utilized to gather global spatial information with channel statistics, as expressed by the following formula:

$$S_{m}=GAP\left(X_{m}^{C}\right)=\frac{1}{H\times W}\sum_{i=1}^{H}\sum_{j=1}^{W}X_{m}^{C}\left(i,j\right),m=1,2$$ (12)

Next, the upper and lower global channel descriptors, *S*_1_ and *S*_2_, are stacked together, and the channel soft attention operation is used to generate the feature importance vector $\beta_{1},\beta_{2}\in {\mathbb{R}}^{C}$, as follows:

$$\beta_{1}=\frac{e^{S_{1}}}{e^{S_{1}}+e^{S_{2}}},\beta_{2}=\frac{e^{S_{2}}}{e^{S_{1}}+e^{S_{2}}},\beta_{1}+\beta_{2}=1$$ (13)

Finally, guided by the feature importance vector $\beta_{1}$ and $\beta_{2}$ , the channel-refined features *X^C^* can be obtained by merging the upper features $X_{1}^{C}$ and the lower features $X_{2}^{C}$ in a channel-wise manner as follows [28]:

$$X^{C}=\beta_{1}\cdot X_{1}^{C}+\beta_{2}\cdot X_{2}^{C}$$ (14)

In summary, by employing CRU, the redundancy of the spatial-refined feature map *X^S^* along the channel dimension is further reduced. Additionally, CRU extracts rich representative features by employing lightweight convolution operations while handling redundant features using cost-effective operations and feature reuse schemes. Subsequent experiments demonstrated that the sequential spatial-channel combination (SRU+CRU) achieved better performance compared to the sequential channel-spatial combination (CRU+SRU), the parallel use of the two units (CRU&SRU), and the individual use of each unit (CRU‖SRU).

#### 3.2.3. Parameters analysis.

The number of parameters for a standard convolution with a kernel size of k×k can be determined using the formula:

$$P_{C}=k\times k\times C_{1}\times C_{2}$$ (15)

In the SCConv module, all parameters are concentrated in the split and transform stages of the CRU. The split stage includes two 1×1 convolutions, and in the transform stage, the number of parameters is mainly concentrated in the group convolution and the two pointwise convolutions. Therefore, the total number of parameters in the SCConv module consists of five parts and can be calculated by the following formula:

$$P_{SC}=1\times 1\times \alpha C\times \frac{\alpha C}{r}+1\times 1\times \left(1-\alpha \right)C\times \frac{\left(1-\alpha \right)C}{r}\;\;+k\times k\times \frac{\alpha C}{rg}\times C\times g+1\times 1\times \frac{\alpha C}{rg}\times C\;\;+1\times 1\times \frac{\left(1-\alpha \right)C}{r}\times \left(C-\frac{1-\alpha }{r}C\right)$$ (16)

where α represents the split ratio, *r* denotes the compression ratio, *g* is the group size of the GWC, and *C*_1_ and *C*_2_ are the input and output feature channels, respectively. Here, his paper provides a contrast to demonstrate the effectiveness of the newly introduced SCConv. In the experiment, the parameter set is $\alpha =\frac{1}{2}$, *r* = 2, *g* = 2, *k* = 3, $C_{1}=C_{2}=C$, the number of parameters can be lowered by a factor of 5, where $P_{C}/P_{SC}\approx 5$, while the module obtains superior performance compared to normal convolution.

### 3.3 Multy-layer feature fusion

**Fig 8:**
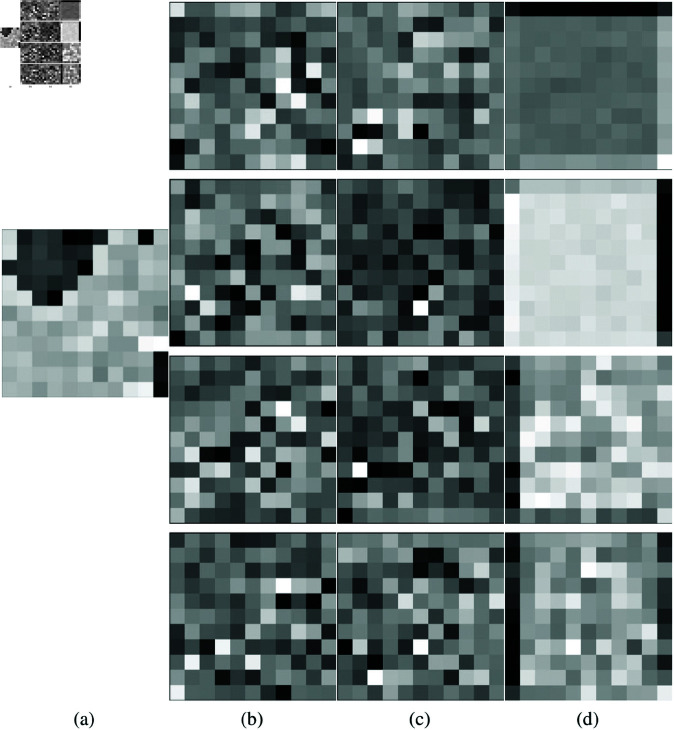
Different depths contain varying quantities of feature information. (a) Feature maps of 1×1 Conv. (b) First SCConv module’s feature maps. (c) Second. (d) Third.

As shown in Fig 8, different depth layers of the proposed SCNet have different levels of feature information. To effectively utilize these different features between SCConv modules, this study proposes an MLFF unit to connect feature maps at different layers. The MLFF unit in the proposed model is used to combine multiple feature maps belonging to different SCConv groups, as shown in Fig 4. Moreover, the MLFF unit can be regarded as a group of skip connections, which have been demonstrated to effectively address the issues of vanishing gradient and exploding gradient. In the realm of computer vision, the concatenation of multi-layer feature maps can achieve better performance. Yuan *et al*. [29] used cascade representation for recovery and achieved outstanding results in the HSI denoising challenge. In the HSI reconstruction model, Zou *et al*. [30] used the multi-layer fusion module to merge the hierarchical information to generate more representative features, which effectively improved the reconstruction accuracy of HSI. The proposed MLFF unit can be expressed as follows:

$$F_{C}=concat\left(F_{2},F_{3},F_{4}\right)$$ (17)

where *F*_2_, *F*_3_, *F*_4_ represent the feature maps of different layers in Fig 8, respectively.

Let *C* represent the number of feature channels, the combined feature $F_{c}\in {\mathbb{R}}^{N\times 3C\times P\times P}$ is restored to its original size by a 1×1 convolutional layer $F_{out}\in {\mathbb{R}}^{N\times C\times P\times P}$

$$F_{out}=W_{C}*F_{c}+b_{C}$$ (18)

where *P* is the feature map size, * represents the convolution operation, and *W*_*C*_ and *b*_*C*_ represent the weight parameter and bias parameter of the later convolution layer, respectively.

## 4 Experiment

To assess the effectiveness of SCNet, the experiments will be conducted using four different data sets. These experiments are designed to compare and confirm the accuracy and efficiency of the proposed network in relation to other methods. Three quantitative metrics, overall accuracy (OA), average accuracy (AA), and Kappa coefficient, were used to measure the accuracy of each method. Specifically, OA represents the proportion of all pixels correctly classified. AA represents the average accuracy over all categories. The Kappa coefficient reflects the agreement between ground-truth and classification results. Higher values of the three measures indicate better classification results. In addition, model parameters (Para), floating-point numbers (FLOPs), and Training time and Testing time are used to evaluate the comprehensiveness of the computational complexity and efficiency model.

### 4.1 Datasets

Pavia University (UP) dataset was acquired by a ROSIS sensor at the University of Pavia in Northern Italy. It contains 115 bands in the spectral region of 430 nm to 860 nm. Due to spectral mixing caused by different materials (such as buildings, roads, and vegetation) appearing within the same pixel in urban environments, the number of bands was reduced to 103 after removing irrelevant bands. The dataset has a spatial resolution of 1.3 m, a spatial size of 610×340, and includes a total of 42,776 labeled pixels categorized into 9 classes.

Kennedy Space Center (KSC) dataset was collected by the AVIRIS instrument over the Kennedy Space Center in Florida. It contains 224 bands in the 400 nm to 2500 nm spectral region. Due to the high correlation among certain bands of the AVIRIS sensor, redundant bands need to be removed to optimize information representation. After eliminating irrelevant bands, a total of 176 bands were retained. The dataset has a spatial resolution of 18m, a spatial size of 512×614, a total of 5211 labeled pixels and 13 categories.

WHU-Hi-LongKou (WHLK) dataset [31] was obtained using a Headwall Nano-Hyperspec imaging sensor with an 8 mm focal length, mounted on a DJI Matrice 600 Pro (DJI M600 Pro) UAV platform [32] in Longkou Town, Hubei Province, China. The study area is a simple agricultural scene, which contains six crop species: corn, cotton, sesame, broad-leaf soybean, narrow-leaf soybean, and rice. It contains 274 bands from 400 to 1000 nm with a spatial dimension of 550×400, a total of 204, 542 labeled pixels and 9 categories, and a spatial resolution of about 0.463m.

IndianPines (IP) dataset was gathered by AVIRIS sensor over the Indian Pines test site in North-western Indian. It contains 200 bands in the 400 nm to 2500 nm spectral region and after removing invalid bands such as those affected by water vapor absorption, it includes 200 bands. The dataset hsa a spatial resolution is 20 m, a spatial size of 145×145, a total of 10249 labeled pixels. There are 16 categories in the image with uneven distribution.

Deep learning algorithms are data-driven and rely on a large number of labeled training samples. The more labeled data that is fed into the training, the higher the accuracy produced. Nevertheless, an increase in data leads to a corresponding increase in the amount of time required and the level of computing complexity. Notably, even with only little training examples, the proposed SCNet continues to operate at an exceptional level. Therefore, the size of training and validation samples in the experiment is set at a minimum level. For UP, 1% of the samples were selected for training and another 1% for validation. Both KSC and IP use a 5% sample size for training and validation. For WHLK, we exclusively choose 0.2% of the samples for training and another 0.2% for validation. Tables 1–4 list the training, validation, and test samples for the four datasets. To mitigate the issue of class imbalance in the IP dataset, we employed an oversampling approach for the minority classes (Classes 1, 7, 9, and 16) to increase their training sample sizes.

**Table 1:** The class names of UP dataset along with the number of training, validation, and test samples for each class.

Order	Class	Total samples	Training samples	Validation samples	Testing samples
1	Asphalt	6631	66	66	6499
2	Meadows	18649	186	186	18277
3	Gravel	2099	20	20	2059
4	Trees	3064	30	30	3004
5	Painted metal sheets	1345	13	13	1319
6	Bare soil	5029	50	50	4929
7	Bitumen	1330	13	13	1304
8	Self-blocking bricks	3682	36	36	3610
9	Shadows	947	9	9	929
	Total	42776	423	423	41930

**Table 2:** The class names of KSC dataset along with the number of training, validation, and test samples for each class.

Order	Class	Total samples	Training samples	Validation samples	Testing samples
1	Scurb	761	38	38	685
2	Willow swamp	243	12	12	219
3	CP hammock	256	12	12	232
4	Slash pine	252	12	12	228
5	Oak/Broadleaf	161	8	8	145
6	Hardwood	229	11	11	207
7	Swamp	105	5	5	95
8	Graminoid marsh	431	21	21	389
9	Saprtina marsh	520	26	26	468
10	Cattail marsh	404	20	20	364
11	Salt marsh	419	20	20	379
12	Mud flats	503	25	25	453
13	Water	927	46	46	835
	Total	5211	256	256	4699

**Table 3:** The class names of WHLK dataset along with the number of training, validation, and test samples for each class.

Order	Class	Total samples	Training samples	Validation samples	Testing samples
1	Corn	34511	69	69	34373
2	Cotton	8374	16	16	8342
3	Sesame	3031	6	6	3019
4	Broad-leaf soybean	63212	126	126	62960
5	Narrow-leaf soybean	4151	8	8	4135
6	Rice	11854	23	23	11808
7	Water	67056	134	134	66788
8	Roads and houses	7124	14	14	7096
9	Mixed weed	5229	10	10	5209
	Total	204542	406	406	203730

**Table 4:** The class names of IP dataset along with the number of training, validation, and test samples for each class.

Order	Class	Total samples	Training samples	Validation samples	Testing samples
1	Alfalfa	46	3	3	40
2	Corn-notill	1428	42	42	1344
3	Corn-mintill	830	24	24	782
4	Corn	237	7	7	223
5	Grass-pasture	483	14	14	455
6	Grass-trees	730	21	21	688
7	Grass-pasture-mowed	28	3	3	22
8	Hay-windrowed	478	14	14	450
9	Oats	20	3	3	14
10	Soybean-notill	972	29	29	914
11	Soybean-mintill	2455	73	73	2309
12	Soybean-clean	593	17	17	559
13	Wheat	205	6	6	193
14	Woods	1265	37	37	1191
15	Buildings-grass-trees-drives	386	11	11	364
16	Stone-steel-towers	93	3	3	87
	Total	10249	307	307	9635

### 4.2 Experiment setting

For this work, to compare the time consumption of training and testing, all experiments were performed on a computer configured with 12 GB memory and NVIDIA GeForce RTX 3060 GPU, and the programming environment is Python-3.8.5 with the PyTorch-1.10.0 framework. The experimental results are shown as the average and standard deviation of 10 experiments.

#### 4.2.1 Split ratio α.

**Fig 9:**
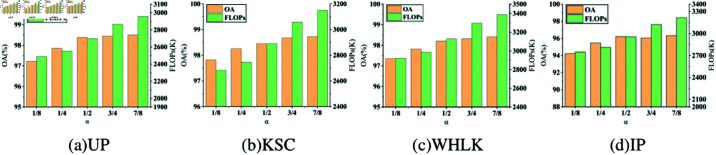
The FLOPs and OA on four datasets with different split rRESEARCHARTICLEatios α in SCConv.

To explore the effect of different split ratio α in CRU module, the split ratio α was adjusted incrementally from 1/8 to 7/8 and evaluated the overall accuracy and FLOPs on four datasets. As shown in Fig 9, the accuracy of SCNet rises with the increase of the split ratio α. The higher α allows the model to capture more comprehensive feature information during the CRU’s transformation phase, thereby improving the model’s overall performance. When α is set to 1/2, the network achieves the best trade-off between FLOPs and accuracy. Consequently, for subsequent experiments, α=1/2 will be chosen as the optimal split ratio for SCConv to ensure a balanced compromise between performance and efficiency.

#### 4.2.2 Patch size selection.

**Fig 10:**
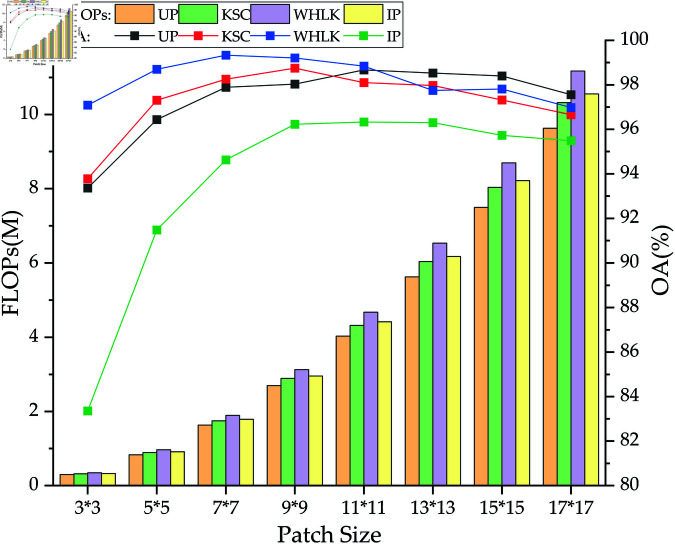
Classification results for varying patch sizes on four datasets.

To utilize spatial information for spectral spatial classification, the 3D cube was utilized as input to the model, which preserves all the bands in the original image in the spectral dimension. Since different cube sizes can impact the HSI classification results, in order to find the best cube size, a series of experiments were conducted in the range of 3,5,7,9,11,13,15,17. Fig 10 shows the overall accuracy of SCNet on four hyperspectral datasets for different spatial sizes. It can be found that as the patch size increases, the classification performance of the model initially increases and then decreases. This is due to the fact that as the space size increases, the model learns more spatial information, so the classification accuracy improves to some extent on the four datasets. However, if the spatial size is above a certain value, the classification accuracy starts to decrease because additional neighboring pixels introduce noise and restrict the model’s ability to extract distinctive features from the center pixel. When the patch size is set to 9, high accuracy is achieved on all four datasets. In addition, the FLOPs of the model gradually rise as the size increases. Therefore, to optimize the classification performance and efficiency of the model, the patch size will be set to 9.

#### 4.2.3 Parameters configuration.

Table 5 provides the implementation details for each layer. It should be noted that all average pools have been implemented using the adaptive average pool, which adapts itself to the input sizes in order to vectorize the multidimensional input array into a 1-D array.

**Table 5:** Network configuration of the SCNet model on Indian Pines dataset.

Layer	Kernel	Str./Pad.	Normalization	Activation	Parameter
Conv_1	32×200×1×1	1/No	No	No	6432
SCConv					
SRU					
	—	—	GN (*g* = 16)	Sigmoid	64
CRU					
Conv_up	8×16×1×1	1/No	No	No	128
Conv_low	8×16×1×1	1/No	No	No	128
GWC	32×8×3×3 (*g* = 2)	1/Yes	No	No	1184
PWC_up	32×8×1×1	1/No	No	No	256
PWC_low	24×8×1×1	1/No	No	No	192
AvgPool					0
SCConv					
SRU					
	—	—	GN (*g* = 16)	Sigmoid	64
CRU					
Conv_up	8×16×1×1	1/No	No	No	128
Conv_low	8×16×1×1	1/No	No	No	128
GWC	32×8×3×3 (*g* = 2)	1/Yes	No	No	1184
PWC_up	32×8×1×1	1/No	No	No	256
PWC_low	24×8×1×1	1/No	No	No	192
AvgPool					0
SCConv					
SRU					
	—	—	GN (*g* = 16)	Sigmoid	64
CRU					
Conv_up	8×16×1×1	1/No	No	No	128
Conv_low	8×16×1×1	1/No	No	No	128
GWC	32×8×3×3 (*g* = 2)	1/Yes	No	No	1184
PWC_up	32×8×1×1	1/No	No	No	256
PWC_low	24×8×1×1	1/No	No	No	192
AvgPool					0
Conv_2	32×96×1×1	1/No	No	No	3104
Conv_3	72×32×3×3	1/No	No	No	20808
FC1	1152	—	BN	ReLU	144
AvgPool					0
FC2	C = 16	—	No	Softmax	18448

#### 4.2.4 Comparison method.

To fully evaluate the effectiveness of SCNet, this paper selected several state-of-the-art deep learning methods for comparison, These include CNN-based (SSRN [20], HybridSN [17], S3Net [22]), Generative Adversarial Network(CA-GAN [33]) and attention-based methods (DBDA [21]). In addition, SVM with RBF kernels has been considered [34]. The patch size of each classification method is determined based on the specifications provided in its original research paper. Then, the above methods are briefly introduced respectively.

SSRN: Spectral and Spatial Residual Network (SSRN) utilizes spectral and spatial residual blocks to continuously learn rich spectral features and spatial context features from hyperspectral images. This is achieved by connecting 3D convolutional layers through identity maps. The input space for SSRN is of size 7×7×c, where *c* represents the number of spectral bands.

HybridSN: HybridSN integrates spectral-spatial 3D-CNN with spatial 2D-CNN to utilize joint feature information and further learn more spatial representations. The size of the input space is 9×9×30.

S3Net: S3Net consists of two lightweight spectral-spatial networks in a dual branch, Each branch consists of 1D-CNN and 2D-CNN to extract spectral-spatial features. The size of the input space is 9×9×60.

CA-GAN: A generative adversarial network based on collaborative learning and attention mechanisms for HSI. The size of the input space is 27×27×20.

DBDA: A two-branch CNN architecture with a dual attention mechanism is used in HSI classification technique to efficiently collect both spatial and spectral data. The size of the input space is 9×9×c.

For SSRN, HybridSN, S3Net, CA-GAN, DBDA, and the proposed method, the batch size is set to 64, the optimizer is set to Adam, the learning rate is 0.0005, and all are trained for about 200 epochs.

### 4.3 Experiment results and discuss

#### 4.3.1 Classification performance.

**Fig 11:**
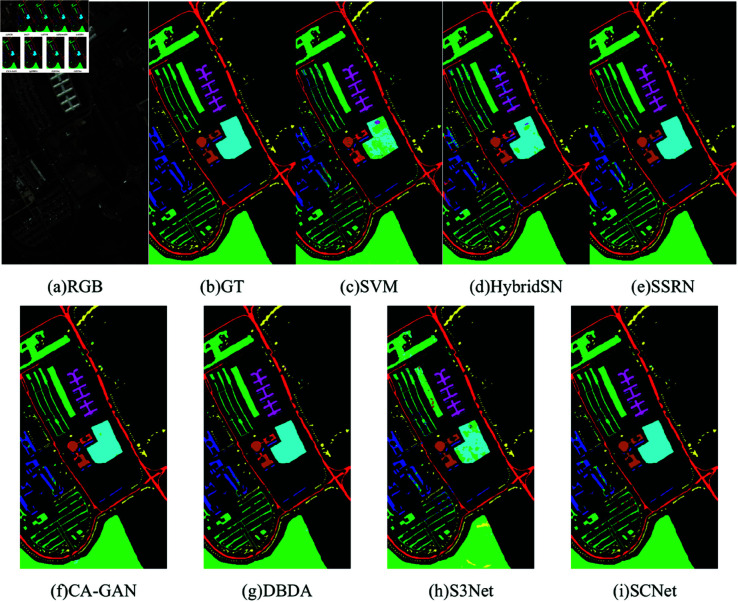
Classification maps for the UP dataset using 1% training samples. (a) RGB image. (b) Ground-truth (GT). (c–h) The classification maps with disparate algorithms.

Table 6 displays the experimental findings of each approach for the UP dataset, including OA, AA, Kappa, and the accuracy of each category. The maximum value for each item is highlighted in bold. In addition, the three-color map of this dataset, the ground-truth map, and the classification map of each method are shown in Fig 11. Because the spatial information is not considered, only the spectral features of the original HSI are used to train the network for classification, which leads to poor classification performance of SVM, and the classification map has a lot of incorrect classifications, and noise-like situations appear in each category. Although HybridSN utilize the spatial and spectral features of HSI, because it cannot learn effective features with limited training samples, the classification effect is poor, and its classification maps produce quite obvious errors. S3Net for limited sample classification has a high OA but a low AA due to the poor categorization of the fifth category, resulting in block errors in the classification map. In general, SSRN, and DBDA exhibit good classification performance and fewer noisy pixels in the classification map, and among these comparison methods, CA-GAN obtains excellent classification performance in most classes by leveraging generated samples with high quality, especially in the classes having fewer samples. Still, their OA and Kappa coefficients are lower than those of our SCNet method. It can be concluded that our proposed method can still extract effective features while reducing channel and spatial redundancy when the training samples are limited, which not only results in a reduction in the number of parameters and computation of the model but also achieves better classification performance.

**Table 6:** The classification results for the UP dataset with 1% training samples.

Class	SVM	HybridSN	SSRN	CA-GAN	DBDA	S3Net	Proposed
1	87.44±8.25	89.77±5.63	99.34±0.35	93.46±5.24	93.54±3.23	96.43±1.56	96.93±0.88
2	89.30±6.31	96.76±1.25	98.69±0.87	98.78±1.58	99.76±0.65	92.97±0.45	99.42±0.29
3	80.26±15.23	73.35±11.54	99.25±0.43	100.0±0.00	99.89±0.58	87.21±3.34	98.62±3.12
4	96.71±5.69	95.33±3.22	100.0±0.00	96.57±2.33	97.65±1.16	95.86±4.25	99.81±1.49
5	92.04±3.45	91.05±6.11	99.92±0.25	99.62±0.76	98.89±0.96	100.0±0.00	100.0±0.00
6	91.70±4.89	95.68±2.85	99.39±0.51	97.75±1.36	99.15±0.97	78.22±18.37	98.83±0.41
7	82.07±5.22	92.70±5.37	96.56±2.14	100.0±0.00	98.93±1.31	98.85±0.72	94.62±0.36
8	78.59±9.65	87.05±8.26	87.05±6.67	91.98±5.12	92.95±3.25	89.65±13.01	90.58±2.22
9	99.89±0.56	96.89±3.68	99.78±0.26	99.55±0.61	99.89±0.33	99.89±0.41	99.77±0.55
OA(%)	88.46±4.32	91.42±6.43	97.83±2.33	97.13±2.35	97.86±1.24	91.96±0.75	98.03±0.30
AA(%)	88.67±5.61	90.59±7.49	97.78±1.89	97.52±2.16	97.84±1.05	93.23±2.15	97.62±0.47
Kappa(×100)	84.37±6.51	88.54±8.66	97.11±0.78	96.18±2.07	97.16±0.26	89.36±3.26	97.39±0.39

**Fig 12:**
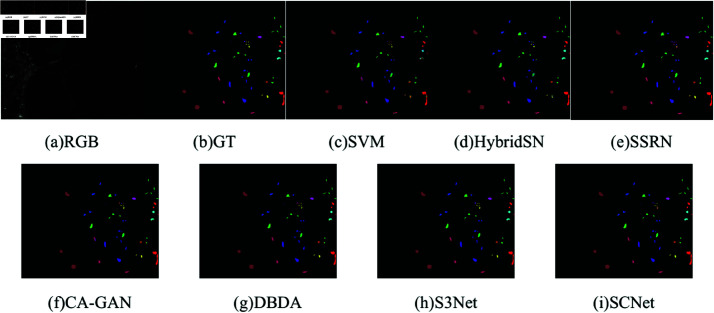
Classification maps for the KSC dataset using 5% training samples. (a) False-color image. (b) Ground-truth (GT). (c–h) The classification maps with disparate algorithms.

Table 7 records the results of all metrics for the KSC dataset. It is obvious that our method achieves the best classification performance compared with all the compared methods, with OA, AA and Kappa exceeding 98%, 97% and 98% respectively. Specifically, due to the limited number of training samples, SVM and HybridSN models have large differences in classification accuracy on different categories. For example, in the first and 13th categories, the classification results of the three methods are all above 91%, but the accuracy is very low in the fourth and fifth categories. Although SSRN, CA-GAN, and DBDA all exhibit excellent OA, they have a comparatively low AA, with less than 83% accuracy on multiple categories. S3Net shows good output, but less than 90% accuracy for more than one class. In contrast, our SCNet demonstrates excellent classification performance, with over 92% accuracy across 13 categories. Fig 12 illustrates the three-color map of the KSC dataset, the ground-truth map, and the classification map of different models. Clearly, the classification maps of the other methods contain significant error points. Our approach yields highly precise classification outcomes with minimizing the amount of misclassified pixels. This also shows that our proposed method can obtain better feature representation with limited training samples, so as to obtain a more accurate classification map.

**Table 7:** The classification results for the KSC dataset with 5% training samples.

Class	SVM	HybridSN	SSRN	CA-GAN	DBDA	S3Net	Proposed
1	92.51±10.46	100.0±0.00	100.0±0.00	100.0±0.00	100.0±0.00	91.49±2.03	100.0±0.00
2	84.74±2.19	93.12±5.83	100.0±0.00	98.97±1.70	100.0±0.00	97.30±5.27	99.06±0.68
3	85.18±4.18	74.21±4.59	98.24±4.70	100.0±0.00	93.65±3.54	100.0±0.00	92.28±0.24
4	67.11±7.63	73.44±6.64	87.42±3.73	82.07±12.67	89.78±2.83	97.84±5.09	93.66±2.58
5	50.97±4.95	79.02±8.50	57.92±2.81	82.17±11.03	91.03±2.71	89.36±3.15	93.01±0.88
6	69.43±2.94	92.82±6.09	96.11±0.41	100.0±0.00	100.0±0.00	100.0±0.00	100.0±0.00
7	76.99±10.11	91.31±3.56	78.26±5.52	75.86±17.64	72.58±6.01	100.0±0.00	93.62±2.32
8	88.58±1.53	87.63±8.19	98.46±1.32	98.22±0.79	98.98±0.79	100.0±0.00	97.24±0.00
9	85.58±3.29	89.17±2.06	100.0±0.00	92.63±2.37	100.0±0.00	98.20±0.92	100.0±0.00
10	88.41±1.85	95.85±6.01	100.00±0.00	100.0±0.00	100.0±0.00	100.0±0.00	100.0±0.00
11	94.73±2.17	98.91±3.58	99.47±0.89	100.0±0.00	100.0±0.00	99.49±1.00	100.0±0.00
12	96.85±1.00	92.71±2.89	99.77±0.62	100.0±0.00	98.04±1.23	88.19±5.62	97.61±0.47
13	99.88±1.74	100.0±0.00	100.0±0.00	100.0±0.00	100.0±0.00	100.0±0.00	100.0±0.00
OA(%)	88.25±0.66	92.87±1.15	96.46±1.02	96.87±2.18	97.89±1.52	96.82±1.03	98.45±0.93
AA(%)	83.15±2.58	89.86±1.57	93.51±1.03	94.61±3.26	95.70±1.20	96.461±1.92	97.42±0.30
Kappa(×100)	86.93±1.19	92.06±1.28	96.07±1.17	96.52±2.51	97.66±0.89	97.07±1.11	98.27±1.01

**Fig 13:**
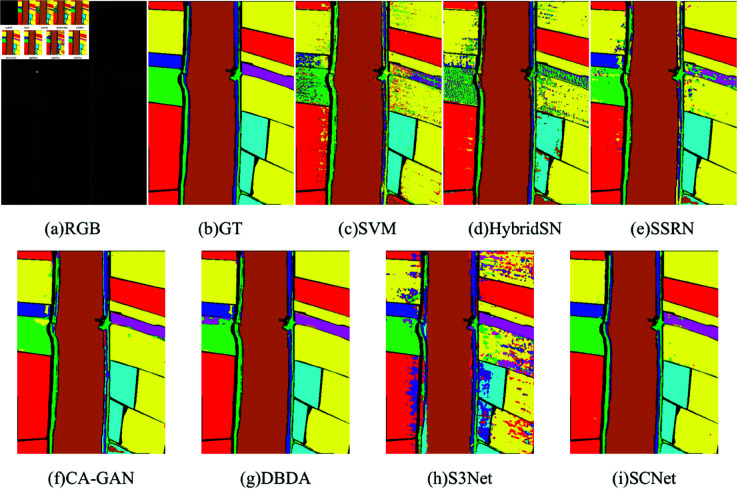
Classification maps for the WHLK dataset using 0.2% training samples. (a) False-color image. (b) Ground-truth (GT). (c–h) The classification maps with disparate algorithms.

The experimental results for the WHLK dataset are shown in Table 8 and Fig 13. Our method still maintains high classification accuracy and performs better than other methods. Similarly, SCNet has more than 90% accuracy in all nine categories, and the results are more balanced. In contrast, more than one category has less than 90% or worse accuracy in all other compared methods. This is because our proposed method can still extract effective features while reducing channel and spatial redundancy, and effectively fuse the features between different layers, so that the feature difference between the same category becomes small, and the phenomenon of “foreign objects with the same spectrum, different spectrum with the same object” is avoided as far as possible, which not only improves the classification accuracy, but also reduces the classification accuracy difference of each category. Moreover, the comparison with the ground truth images can prove that the classification maps generated by our method are the most accurate and smooth. The precise classification of each ground object is crucial for practical applications, and SCNet has great potential and advantages in achieving this goal.

**Table 8:** The classification results for the WHLK dataset with 0.2% training samples.

Class	SVM	HybridSN	SSRN	CA-GAN	DBDA	S3Net	Proposed
1	95.89±4.46	98.01±1.96	99.16±3.02	98.78±2.63	99.99±2.69	90.04±2.59	99.29±0.29
2	58.32±1.34	52.30±0.80	76.94±1.40	94.12±1.44	98.29±0.87	92.09±4.03	96.39±0.88
3	86.43±8.99	27.37±3.59	98.73±5.07	100.0±0.00	93.88±9.58	93.42±6.35	100.0±0.00
4	84.91±1.63	91.11±3.16	93.03±2.91	97.85±14.34	98.69±0.46	76.27±3.20	99.57±0.21
5	39.46±23.62	28.87±0.42	85.67±2.83	96.19±1.15	77.680.35	97.65$\omega_{cd}$0.13	99.53${\text{CD}}_{\text{V}}$0.41
6	92.61$r_{CD_{V}}$1.75	94.73$r_{I_{1}}$3.54	96.96$r_{V_{1}}$4.26	89.13$\omega_{v}$0.83	99.88${\vec{V}}_{1}$2.38	68.09$r_{V_{1}}$1.35	99.91$\omega_{m}$0.00
7	98.89$\vec{M}$0.10	93.51$\omega_{cd}$5.03	97.83${CD}_{V}$6.47	99.23$r_{CD_{V}}$0.38	99.24${\vec{CD}}_{V}$4.22	94.85$r_{V_{1}}$0.25	99.74±0.13
8	70.24±3.08	76.33±2.21	76.63±1.95	84±16.19	81.13±3.25	58.27±3.77	90.83±2.22
9	55.31±4.01	68.34±5.55	67.13±10.15	75.86±6.57	88.64±1.22	85.58±0.88	98.01±1.49
OA(%)	88.98±0.80	88.51$\omega_{cd}$0.48	94.25$\omega_{cd}$1.11	96.96${\vec{CD}}_{V}$1.62	97.69$r_{V_{1}}$0.73	85.16${\vec{CD}}_{V}$1.98	99.21$r_{{\hat{V}}_{2}}$0.30
AA(%)	75.78${\vec{\hat{V}}}_{2}$2.58	70.06$r_{I_{2}}$1.08	88.01$r_{V_{2}}$1.86	92.79$\vec{M}$2.56	93.05$r_{V_{2}}$1.22	84.03${\vec{CD}}_{V}$2.25	98.21$r_{{\hat{V}}_{1}}$0.47
Kappa(×100)	85.34±1.19	84.75±0.65	92.39±1.49	95.98$\omega_{v}$2.15	96.96$\omega_{m}$0.97	81.04±1.89	98.97±0.39

**Fig 14:**
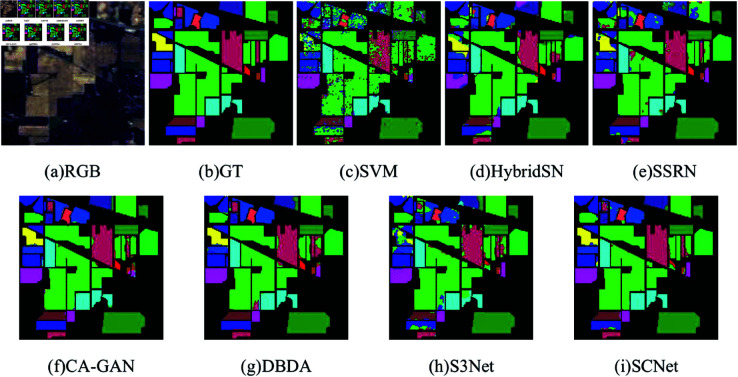
Classification maps for the IP dataset using 5% training samples. (a) False-color image. (b) Ground-truth (GT). (c–h) The classification maps with disparate algorithms.

Table 9 shows the classification results of different methods on the IP dataset, and the classification graphs of different methods and ground truth are shown in Fig 14. Since the IP dataset has inter-class imbalance, the classification performance of most compared methods is limited. SVM only considers spatial domain information, so the classification effect is poor, and the noise in the classification graph is serious. Although HybridSN comprehensively consider the spatial spectrum information of hyperspectral images, there are still obvious misclassifications for the 1st, 7th, and 16th categories with fewer training samples. The classification performance of SSRN and DBDA based on the attention mechanism is relatively good, but there are still poor classification results for individual categories. CA-GAN using generative adversarial technology and S3Net using sample pairs as training input have achieved 98.12% and 98.57% for the classification of the 16th category, which shows the superiority of these two technologies in the case of small sample classification. The classification accuracy of the SCNet method proposed by us has reached 100%, 100%, and 98.42% for the 1st, 7th, and 16th categories, which also proves the effectiveness of SCNet in the case of limited training samples.

**Table 9:** The classification results for the IP dataset with 5% training samples.

Class	SVM	HybridSN	SSRN	CA-GAN	DBDA	S3Net	Proposed
1	30.40±15.56	77.76±12.97	86.24±6.95	95.51±4.55	87.51±0.13	100.0±0.00	100.0±0.00
2	52.51±1.33	80.56±3.30	91.43±4.32	96.42±2.13	94.27±1.32	84.73±5.87	94.25±2.03
3	55.33±8.87	85.12±4.91	92.41±4.52	96.58±2.33	97.21±2.58	82.21$\sigma_{m_{F}}$2.87	94.36$\sigma_{{cd}_{F}}$1.70
4	38.24$\sigma_{v_{P}}$7.51	88.02$\sigma_{m_{P}}$7.54	85.74$\sigma_{{cd}_{P}}$6.63	95.07$\sigma_{v_{O}}$4.71	97.29±5.58	81.69±1.08	97.79±2.08
5	82.18±7.13	92.62±3.36	98.89±0.95	96.14±4.05	99.31±7.12	98.18±4.05	98.22±0.56
6	81.13±3.49	95.02±1.43	98.61±0.67	99.65±0.43	98.17±1.26	91.29±2.69	98.57±0.54
7	45.46±23.48	86.69±15.17	79.16±9.53	94.99±0.12	68.75±2.33	100.0±0.00	100.0±0.00
8	89.76±2.27	95.98±2.97	98.29±2.44	100.0±0.00	100.0±0.00	95.01±4.82	96.65$\vec{M}$1.11
9	33.81${\vec{V}}_{1}$13.85	40.65${\vec{CD}}_{V}$15.51	86.71$\vec{E}$9.22	55.73$\alpha_{v}$9.85	90.68$\alpha_{m}$5.71	90.91$\alpha_{cd}$3.23	99.57$\omega_{v}$2.26
10	58.07$\omega_{m}$5.78	89.20$\omega_{cd}$5.47	89.46$\alpha_{v}$2.19	98.66$\alpha_{m}$0.35	84.82$\vec{E}$0.35	89.25$\omega_{v}$5.67	94.27$\omega_{m}$1.20
11	58.32$\alpha_{cd}$5.25	91.49±2.12	89.99±3.73	99.75±0.21	99.49±0.21	90.37±3.33	98.11±0.85
12	41.67±7.67	84.38±8.54	83.95±2.13	92.37±3.42	96.44±1.32	96.67±4.35	93.39±2.13
13	84.59±6.04	87.32±10.46	99.23±0.76	97.96±1.68	98.01±0.72	100.0±0.00	99.86±0.23
14	85.36±3.36	93.12±4.12	95.44±2.08	95.71±0.42	94.83±1.05	92.95±2.03	96.53±1.03
15	60.00±9.48	82.49±9.61	89.45±5.41	92.36${\vec{V}}_{1}$1.08	97.53${\vec{CD}}_{V}$0.93	97.89$\vec{M}$3.65	94.88${\vec{V}}_{2}$1.32
16	98.41$\omega_{v}$2.38	79.89$\omega_{m}$7.53	92.76$\omega_{cd}$2.91	98.12$\omega_{v_{C}}$1.16	98.83$\omega_{m_{C}}$0.56	98.57$\omega_{{cd}_{C}}$1.24	98.42$\omega_{v}$0.81
OA(%)	63.89±2.33	88.33±1.48	91.94±0.15	95.63±0.51	95.71±2.51	89.89±1.13	96.23±1.00
AA(%)	62.21±2.46	84.39±2.10	91.11±0.15	95.23±2.27	92.53±3.58	92.48±0.50	96.56±0.85
Kappa(×100)	58.112.98	86.65$\sigma_{m_{F}}$1.69	90.79$\sigma_{{cd}_{F}}$1.75	97.01$\sigma_{v_{P}}$0.63	95.12$\sigma_{m_{P}}$0.19	88.41$\sigma_{{cd}_{P}}$1.27	95.69$\sigma_{v_{O}}$0.72

#### 4.3.2 Model complexity analysis.

Similarly, Table 10 records the model complexity, training time, and testing time, demonstrating that the proposed SCNet achieves a highly lightweight design in terms of parameter count and FLOPs. On the UP dataset, SCNet requires only 43,425 parameters and 2.697 FLOPs to achieve an overall accuracy (OA) of 98%, whereas other comparison methods typically require 5-10 times more parameters and 10-70 times more FLOPs to reach a similar accuracy. Moreover, some of these methods still exhibit significantly lower classification performance than SCNet. This indicates that our approach effectively reduces memory usage and initialization costs, making it particularly suitable for devices with limited storage resources. However, the actual testing time does not fully align with the theoretical advantage in FLOPs. This discrepancy primarily arises from the model’s use of 1$\sigma_{m_{O}}$1 convolutions and lightweight operations (such as GWC) to reduce parameter count, which alters the computational pattern. Additionally, on GPUs, standard 3$\sigma_{{cd}_{O}}$3 convolutions typically benefit more from cuDNN optimizations, whereas lightweight operations like GWC may not fully exploit the parallel computing capabilities of the hardware. As a result, although SCNet demonstrates a clear advantage in theoretical computational efficiency, its actual testing time does not decrease proportionally with FLOPs. Nevertheless, in terms of testing speed, SCNet is not inferior to other comparison methods. Therefore, in resource-constrained environments, this approach remains highly practical and efficient.

**Table 10:** Parameter, FLOPs and runing time(s) of different methods for the four data sets.

Datasets	Metrics	SVM	HybridSN	SSRN	CA-GAN	DBDA	S3Net	Proposed
UP	Parameter(K)		1748.217	216.537	320.686	202.751	238.955	43.425
	FLOPs(M)		28.836	81.212	146.509	68.978	33.528	2.697
	Training time(s)	4.756	492.979	57.74	99.95	89.45	53.538	57.547
	Testing time(s)	3.446	130.957	9.526	18.971	18.871	9.266	10.478
KSC	Parameter(K)		3094.269	327.229	536.042	338.187	391.671	50.373
	FLOPs(M)		51.581	139.089	215.338	94.792	57.007	2.891
	Training time(s)	2.491	503.714	45.501	89.801	77.497	109.438	34.612
	Testing time(s)	0.392	24.608	1.618	3.391	3.302	1.428	1.249
WHLK	Parameter(K)		4826.361	471.513	808.534	509.159	583.019	48.769
	FLOPs(M)		80.864	214.65	333.841	146.415	86.685	3.129
	Training time(s)	20.676	225.754	265.249	531.175	449.337	108.775	148.925
	Testing time(s)	20.661	148.226	128.647	278.856	270.575	79.075	136.364
IP	Parameter(K)		3537.024	363.993	1224.592	382.326	424.642	54.600
	FLOPs(M)		59.057	158.382	786.317	107.972	70.156	2.956
	Training time(s)	20.153	1005.7693	266.64	712.925	75.185	106.322	66.143
	Testing time(s)	0.662	71.737	15.022	10.398	8.064	2.625	2.643

Since SVM is a pixel-based model, it takes less time than 3D cube-based models in most cases. Our model preserves all bands of the network input, resulting in an increase in parameters and FLOPs in the WHLK dataset compared to the UP dataset. Nevertheless, SCNet still maintains efficient training and testing speeds for all pixels of this image, demonstrating good hardware adaptability. Furthermore, the model complexity still outperforms other algorithms. Despite the input patch sizes of HybridSN, SSRN, CA-GAN, DBDA, and S3Net being the same or even smaller than ours, SCNet still has the smallest FLOPs. Although the testing times for HybridSN, SSRN, and S3Net on the WHLK dataset are comparable to our method, our method achieves higher accuracy. This means our method better balances accuracy and efficiency.

#### 4.3.3 Analysis of proportion of training samples.

**Fig 15:**
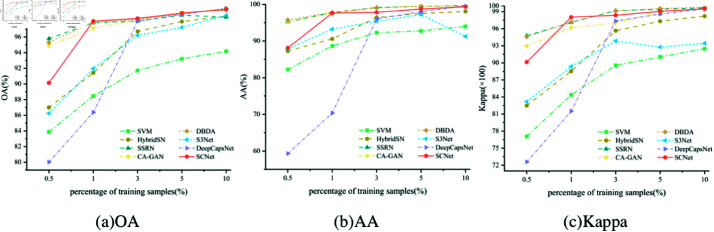
Accuracy of different methods with different numbers of training samples on UP. (a) OA; (b) AA; (c) Kappa.

As mentioned before, deep learning is a data-driven algorithm that heavily relies on a substantial amount of high-quality labeled datasets. Consequently, this part explores the effect of different proportions of training samples on the classification results of the UP datasets, as shown in Fig 15. As anticipated, the accuracy demonstrates improvement as the number of training samples increases. All deep learning-based comparison approaches and our proposed framework exhibit near-perfect performance when provided with sufficient samples, approximately 10% of the entire dataset. Moreover, as the number of training samples increases, the performance disparity between different models diminishes. However, even in cases where there are insufficient samples available for training, our method consistently outperforms other approaches. Given that labeling datasets incur significant costs in terms of manpower and resources, our proposed method offers potential savings.

### 4.4 Ablation studies

To verify the effectiveness of sequential spatial channel combination (SRU+CRU), we design sequential channel space (CRU+SRU), parallel use of two units (CRU${\vec{V}}_{1}$SRU), and separate use of two units (CRU$\vec{M}$SRU), and compare the performance of these four combinations with sequential spatial channel combination. As can be seen in Table 11, the sequential spatial channel combination outperforms the other four combinations on the UP dataset, whether OA, AA or Kappa. Therefore, the sequential space composition (SRU+CRU) strategy was adopted to compose SCConv to further improve the model performance.

**Table 11:** Ablation experimental results of different sequential spatial channel combinations on the UP dataset.

Module	OA (%)	AA (%)	Kappa (${\vec{CD}}_{V}$100)	FLOPs (M)	Para (K)	Testing time (s)
SRU	95.11	94.68	93.46	2.230	37.761	6.584
CRU	94.86	94.65	93.14	2.697	43.425	9.752
SRU&CRU	94.85	94.64	93.13	2.697	43.425	14.094
CRU+SRU	95.36	95.02	93.82	2.697	43.425	11.812
SRU+CRU	98.03	97.62	97.39	2.697	43.425	10.478

To assess the validity of ZCA, we respectively employed PCA to select 20, 40, 60 spectral bands for the ablation experiments. The experimental results are presented in Table 12. From the results, it can be observed that using ZCA to retain all band information significantly enhances the classification performance compared to using PCA to select the principal bands, which fully demonstrates its validity and superiority. Although more parameters and computational efforts are introduced, the increase is relatively minor and acceptable.

**Table 12:** Ablation experimental results of different numbers of channels on the UP dataset.

Band	OA(%)	AA(%)	KAPPA($\vec{E}$100)	Para(K)	FLOPs(M)
PCA:20	96.24	95.99	94.99	40.769	2.482
PCA:40	96.18	96.01	94.92	41.409	2.533
PCA:60	95.52	95.59	94.71	42.049	2.585
PCA:80	94.98	94.73	94.03	42.689	2.637
ZCA:103	98.03	97.62	97.39	43.425	2.697

**Fig 16:**
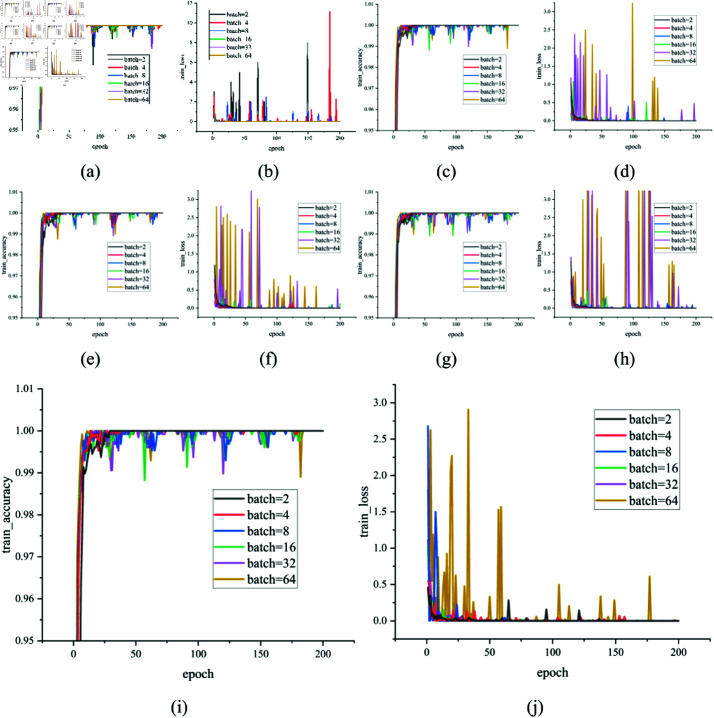
The training accuracy and loss curves of SRU using different normalization methods on the UP dataset. (a,b) BN. (c,d) LN. (e,f) IN. (g,h) SN. (i,j) GN.

Furthermore, to validate the hypothesis that normalization methods other than BN are more stable under smaller batch sizes and to further explore their impact on the model, we replaced GN with BN, LN, and IN in the SRU and recorded the training accuracy and loss curves on the UP dataset for batch sizes of {2, 4, 8, 16, 32, 64}, as shown in Fig 16. From the figure, it can be observed that when the batch size is 2, 4, 8, and 16, the curves corresponding to GN, LN, and SN converge faster, whereas the BN curves exhibit significant oscillations and lack smoothness. In contrast, when the batch size is 32 or 64, the BN curves become smoother and converge by the 50th epoch, while the GN, LN, and SN curves converge more slowly and exhibit oscillations. This behavior can be attributed to the fact that LN, IN, SN, and GN share a similar normalization computation approach, as they are all batch-independent normalization methods. Unlike BN, which relies on cross-sample statistics for computing the mean and variance, these methods normalize based on individual samples or subsets of features, making them more stable in small-batch training or scenarios with significant distribution variations.

As shown in Table 13, training with smaller batch sizes requires more time. This is because, during each backpropagation step, model weights are updated based on the loss of the current batch. A smaller batch size results in more frequent parameter updates, leading to an increase in overall training time. Although replacing GN with LN, IN, or SN also provides good stability and performance, the training time is slightly longer compared to using GN. This is because LN and IN involve more fine-grained computations when calculating the mean and variance, whereas GN, benefiting from its grouped normalization approach, is often more efficient under optimizations provided by modern deep learning libraries such as cuDNN. Additionally, SN requires weighted computations across different normalization methods, further increasing computational complexity.

**Table 13:** The training time of SRU using different normalization methods on the UP dataset.

Batch size	2	4	8	16	32	64
BN training time(s)	1841.647	1426.010	745.464	355.510	85.178	47.648
GN training time(s)	1152.309	593.515	318.329	57.547	43.233	35.854
LN training time(s)	1526.371	598.238	320.375	150.476	84.258	47.154
IN training time(s)	1181.176	611.545	321.771	159.712	89.054	49.514
SN training time(s)	3466.946	1759.417	905.794	442.699	233.374	124.362

Therefore, considering both normalization stability and computational efficiency, we selected GN as the normalization method and set the batch size to 16 as the training standard, achieving a balance between shorter training time and better model stability.

## 5 Conclusion

This paper proposes SCNet, a novel lightweight model for hyperspectral image (HSI) classification. Firstly, unlike most classification methods that employ PCA dimensionality reduction to process the original 3D pixel data, this paper utilizes ZCA whitening operation to not only preserve all band features but also maintain the transformed data closer to the original input data. Additionally, by introducing Spatial Reconstruction Unit (SRU) and Channel Reconstruction Unit (CRU), they can reduce space and channel redundancy in the convolutional layer while implementing a lightweight strategy. Through ablation experiments, it is demonstrated that arranging SRU and CRU sequentially to form SCConv blocks enables optimal performance of our model. Moreover, considering the multi-level disparity of feature maps at different depth layers, MLFF unit is designed to aggregate hierarchical information for generating more representative features. Our approach exhibits several advantages over other state-of-the-art deep learning algorithms and lightweight networks including enhanced classification performance and improved model efficiency.

Furthermore, by conducting comparative experiments, we demonstrate the efficacy and superiority of the proposed SCNet. However, certain areas for improvement have also been identified. For instance, our testing time does not exhibit a comparable advantage to FLOPs in the comparison. This discrepancy may be attributed to frequent data accesses and memory transitions that potentially hinder the computational pipeline from achieving its utmost performance as sample size increases. Consequently, future work will focus on integrating hardware architecture to optimize our approach further and minimize model testing time while identifying a network better suited for hardware.
